# Incidence, risk factors and consequences of preterm birth – findings from a multi-centric observational study for 14 months in Nepal

**DOI:** 10.1186/s13690-020-00446-7

**Published:** 2020-07-17

**Authors:** Abhishek Gurung, Johan Wrammert, Avinash K. Sunny, Rejina Gurung, Netra Rana, Yuba Nidhi Basaula, Prajwal Paudel, Amrit Pokhrel, Ashish KC

**Affiliations:** 1 Golden Community, Lalitpur, Nepal; 2 Department of Women’s and Children’s Health, Uppsala University, 75237 Uppsala, Sweden; 3 Lumbini Provincial Hospital, Government of Nepal, Butwal, Nepal; 4 Bharatpur Hospital, Government of Nepal, Chitwan, Nepal; 5 Ministry of Health and Population, Government of Nepal, Kathmandu, Nepal; 6 Syangya District Hospital, Government of Nepal, Syangya, Nepal

**Keywords:** Preterm, Risk factors, Stillbirth, Neonatal mortality, Nepal

## Abstract

**Background:**

Preterm birth is a worldwide epidemic and a leading cause of neonatal mortality. In this study, we aimed to evaluate the incidence, risk factors and consequences of preterm birth in Nepal.

**Methods:**

This was an observational study conducted in 12 public hospitals of Nepal. All the babies born during the study period were included in the study. Babies born < 37 weeks of gestation were classified as preterm births. For the association and outcomes for preterm birth, univariate followed by multiple regression analysis was conducted.

**Results:**

The incidence of preterm was found to be 93 per 1000 live births. Mothers aged less than 20 years (aOR 1.26;1.15–1.39) had a high risk for preterm birth. Similarly, education of the mother was a significant predictor for preterm birth: illiterate mothers (aOR 1.41; 1.22–1.64), literate mothers (aOR 1.21; 1.08–1.35) and mothers having basic level of education (aOR 1.17; 1.07–1.27). Socio-demographic factors such as smoking (aOR 1.13; 1.01–1.26), use of polluted fuel (aOR 1.26; 1.17–1.35) and sex of baby (aOR 1.18; 1.11–1.26); obstetric factors such as nulliparity (aOR 1.33; 1.20–1.48), multiple delivery (aOR 6.63; 5.16–8.52), severe anemia during pregnancy (aOR 3.27; 2.21–4.84), antenatal visit during second trimester (aOR 1.13; 1.05–1.22) and third trimester (aOR 1.24; 1.12–1.38), < 4 antenatal visits during pregnancy (aOR 1.49; 1.38–1.61) were found to be significant risk factors of preterm birth. Preterm has a risk for pre-discharge mortality (10.60; 9.28–12.10).

**Conclusion:**

In this study, we found high incidence of preterm birth. Various socio-demographic, obstetric and neonatal risk factors were associated with preterm birth. Risk factor modifications and timely interventions will help in the reduction of preterm births and associated mortalities.

**Trial registration:**

ISRCTN30829654.

## Background

Preterm birth (< 37 weeks of gestation) is one of the leading causes of neonatal morbidity and mortality and a significant public health burden [1, 2]. Every year, there are 15 million (11.1%) preterm births of all births worldwide, and 13.3% of these births occur in South Asia alone [1]. In Nepal, it is reported that around 81,000 newborns are born preterm every year [3]. A study conducted by Lee and colleagues in 2010 reported 14% preterm births [4]. Despite the increase in burden of preterm births worldwide, the data available from developing countries like Nepal is very scarce [5]. In order to achieve the Sustainable Development Goal 3 target of reaching the neonatal mortality rate to 12 per 1000 live birth by 2030, it is critical to address the burden of preterm births [6].

Babies born preterm have a higher risk of dying as reported from a multi-country study conducted in low- and middle-income countries (LMICs) [7]. The greater risk of dying has been mostly associated with neonatal infections [8]. In comparison to term infants, they are more prone to short and long-term neurocognitive and motor impairments together with increased risk of malnutrition, chronic diseases and early deaths [9, 10].

Several factors have been identified as risks for preterm birth. Socio-demographic factors such as ethnicity, older age of mothers and smoking have been reported as risk factors for preterm birth [11, 12]. Low education levels of mothers have also been documented as risk factors for preterm birth by many studies [13–15]. Primiparity has been linked as an obstetric risk factor for preterm birth [16]. Further, poor access to antenatal care services during pregnancy leads to poor pregnancy outcomes like preterm births as demonstrated by a hospital-based study in Nepal [17].

Studies have also been conducted showing provider-initiated interventions like induction of labor and caesarean section are attributable to preterm births [5, 18, 19]. However, very few studies have been conducted assessing the risk factors with preterm births in the context of Nepal. In order to develop suitable interventions for preventing morbidities and mortalities associated with preterm, it is essential to understand the underlying risk factors linked with preterm births and manage them [20]. This study is aimed to address this evidence gap through evaluating the incidence, risk factors and consequences related to preterm births in Nepal.

## Method

### Aim

The study aimed to evaluate the incidence, risk factors and consequences of preterm birth in Nepal.

### Study design and setting

This observational study is nested within the Helping Babies Breathe Quality Improvement Project in 12 hospitals of Nepal [21]. These hospitals are government-funded providing referral level obstetric and neonatal care services. The hospitals were selected based on having an annual delivery of > 1000 per year and which were operated by the government. The hospitals were selected randomly for the study, after which the hospitals were divided into four wedges, with each wedge having a large-sized (> 5000 deliveries per year), mid-sized (3–5000 deliveries per year) and small-sized hospitals (> 1000 deliveries per year) [21]. This study was conducted for a period of 14 months from 1 July 2017 to 29 August 2018.

### Study participants

#### Inclusion criteria

All babies who were delivered in the selected hospitals were included for this study.

#### Exclusion criteria

The mothers of the newborn babies who did not provide consent were excluded.

### Data collection and management

A data surveillance system was established in all the hospitals to collect data on babies and their mothers. It included data collectors based in each of the hospitals with data coordinators, who collected data on all pregnant women from admission until discharge through extraction and exit interviews. All pregnant women who were admitted to the hospital for delivery and who consented to data collection were given unique identification (ID) numbers. Data was collected during labor, delivery and post-partum period. Study IDs were allocated for each hospital, which were assigned by the data collectors for each individual pregnant woman. For obstetric information, data were extracted from patient file and Maternity Register using a data retrieval form (Additional file 1). For sociodemographic variables, data were collected through semi-structured interviews with mothers before discharge (Additional file 2).

The forms that were completed were then assessed by the data coordinator at the hospital for completeness and those completed were indexed. The data entry and management team then sorted and indexed the forms and reassessed for completeness. The data were entered and cleaned in Census and Survey Processing System (CSPro). The cleaned data were exported into Statistical Package for the Social Sciences (SPSS) for further data analysis.

### Variables

#### Outcome variable

Preterm births – Babies born before 37 weeks of gestation.

Pre-discharge mortality – The death of newborn before discharge.

#### Demographic variables

Maternal age, ethnicity, education, smoking history and sex of the baby were included.

#### Antenatal variables

Antenatal care (ANC) visit, time of ANC visit and severe anemia during pregnancy were included.

#### Intrapartum variables

Parity, induction of labor, mode of delivery, multiple deliveries, major malformation and were included.

### Statistical analysis

The incidence of preterm births was calculated. For socio-demographic and obstetric characteristics, binary logistics regression was performed to analyze the level of association with preterm births. *P*-value of < 0.05 was considered to be significant. Missing values were excluded from the analysis. Multivariable regression analysis was done for variables that were significant in univariate analysis.

## Results

**Fig. 1:**
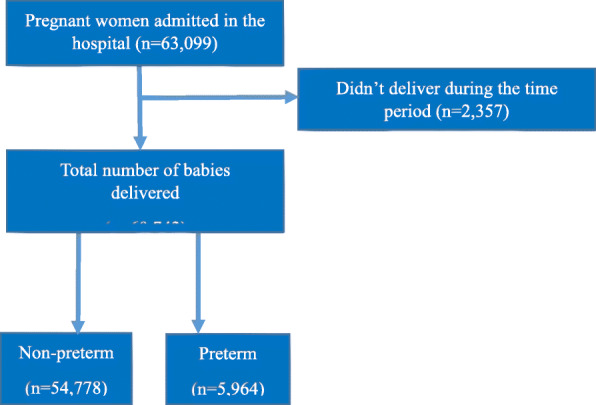
STROBE flow diagram of the study participants

**Fig. 2:**
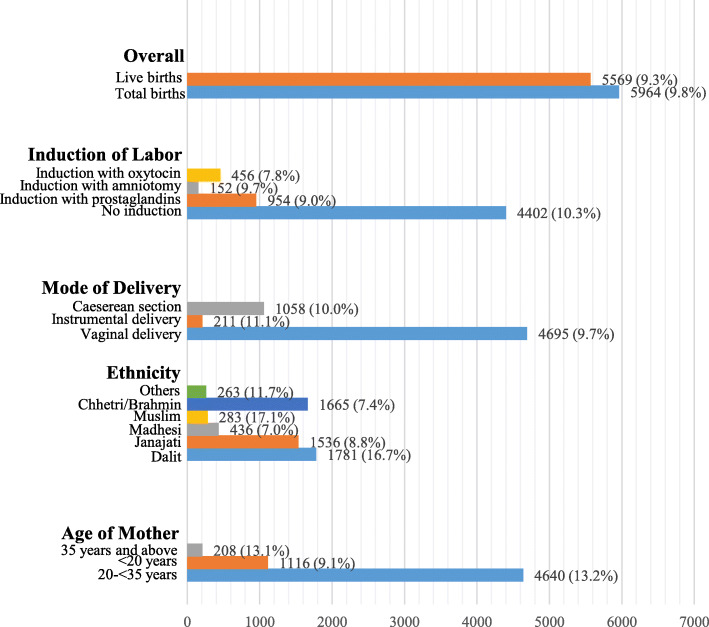
Incidence of preterm birth among the study participants (*n* = 60,742)

A total of 63,099 women were admitted and 60,742 deliveries were conducted during the study period. Among the deliveries, 54,778 were term babies while 5964 babies were born preterm (Fig. 1). The incidence of preterm births was found to be 98 per 1000 total births and 93 per 1000 live births (Fig. 2).

In univariate analysis, socio-demographic characteristic such as age of mother, education level, smoking history and type of fuel all showed significant association (*p* < 0.001) with preterm births. Similarly, obstetric characteristics such as multiple delivery, primiparity, time of first ANC visit, frequency of ANC visits, major malformation and severe anemia during pregnancy were found to be significantly associated with preterm births. Female babies were significantly associated with preterm births (Table 1).

**Table 1:** Socio-demographic, obstetric and neonatal characteristics of preterm and non-preterm babies

Variables	Preterm (***n*** = 5964)	Non-Preterm (***n*** = 54,778)	Total (***n*** = 60,742)	***p***-value	OR (95% CI)
**Age of mother**	23.53 ± 4.7	23.96 ± 4.33			
20- < 35 years	4640 (77.8%)	46,077 (84.1%)	50,717 (83.5%)	< 0.001	Ref
< 20 years	1116 (18.7%)	7322 (13.4%)	8438 (13.9%)	< 0.001	1.51 (1.41–1.62)
> 35 years	208 (3.5%)	1379 (2.5%)	1587 (2.6%)	< 0.001	1.50 (1.29–1.74)
**Level of education (*n*** **= 50,424)**					
Secondary and above	2587 (60.3%)	31,743 (68.8%)	34,330 (68.1%)	< 0.001	Ref
Illiterate	352 (8.2%)	2186 (4.7%)	2538 (5.0%)	< 0.001	1.98 (1.75–2.23)
Literate	572 (13.3%)	4763 (10.3%)	5335 (10.6%)	< 0.001	1.47 (1.34–1.62)
Basic education	776 (18.1%)	7445 (16.1%)	8221 (16.3%)	< 0.001	1.28 (1.18–1.39)
**Smoking (*n*** **= 50,422)**					
No	3664 (85.5%)	41,071 (89.0%)	44,735 (88.7%)		Ref
Yes	622 (14.5%)	5065 (11.0%)	5687 (11.3%)	< 0.001	1.38 (1.26–1.51)
**Type of fuel (*n*** **= 50,209)**					
Clean	2886 (67.6%)	34,628 (75.4%)	37,514 (74.7%)		Ref
Polluted	1384 (32.4%)	11,311 (24.6%)	12,695 (25.3%)	< 0.001	1.47 (1.37–1.57)
**Sex of the baby**					
Boy	2962 (49.7%)	29,788 (54.4%)	32,570 (53.9%)		Ref
Girl	3002 (50.3%)	24,990 (45.6%)	27,992 (46.1%)	< 0.001	1.21 (1.15–1.27)
**Multiple delivery**					
No	5786 (97.0%)	54,576 (99.6%)	60,362 (99.4%)		Ref
Yes	178 (3.0%)	202 (0.4%)	380 (0.6%)	< 0.001	8.31 (6.78–10.19)
**Parity**					
Multipara	1084 (18.2%)	9325 (17.0%)	10,409 (17.1%)	< 0.001	Ref
Nullipara	3168 (53.1%)	26,647 (48.6%)	29,815 (49.1%)	0.55	1.02 (0.95–1.10)
Primipara	1712 (28.7%)	18,806 (34.3%)	20,518 (33.8%)	< 0.001	0.78 (0.72–0.85)
**ANC visit during pregnancy (*n*** **= 49,898)**					
< 4 visits	1362 (32.2%)	10,199 (22.3%)	11,561 (23.2%)	< 0.001	1.65 (1.54–1.77)
≥ 4 visits	2867 (67.8%)	35,470 (77.7%)	38,337 (76.8%)		Ref
**Time of first ANC visit (*n*** **= 49,898)**					
First trimester	1537 (36.3%)	19,316 (42.3%)	20,853 (41.8%)	< 0.001	Ref
Second trimester	1818 (43.0%)	19,105 (41.8%)	20,923 (41.9%)	< 0.001	1.20 (1.11–1.28)
Third trimester	874 (20.7%)	7248 (15.9%)	8122 (16.3%)	< 0.001	1.52 (1.39–1.65)
**Major malformation**					
No	5953 (99.8%)	54,742 (99.9%)	60,695 (99.9%)		Ref
Yes	11 (0.2%)	36 (0.1%)	47 (0.1%)	0.003	2.81 (1.43–5.52)
**Severe anaemia during pregnancy**					
No	5915 (99.2%)	54,642 (99.8%)	60,557 (99.7%)		Ref
Yes	49 (0.8%)	136 (0.2%)	185 (0.3%)	< 0.001	3.33 (2.40–4.62)
**Outcome (*n*** **= 60,062)**					
**Pre-discharge mortality**	460 (50.1%)	459 (49.9%)	919 (1.5%)	< 0.001	10.60 (9.28–12.10)

Half the total pre-discharge mortalities (50.1%) were due to preterm births and its complications. Similarly, preterm births had 11-times more risk of pre-discharge mortality (OR 10.60; 9.28–12.10) (Table 1).

The significant variables in univariate were taken for multivariable regression analysis. In comparison to mothers aged 20–35 years, the risk of preterm birth was almost 1.3 times higher (aOR 1.26; 1.15–1.39) when compared to mothers less than 20 years of age. For mothers above 35 years, the risk was 1.2 times higher (aOR 1.20; 0.98–1.47), however the association was found not significant. In comparison to mothers with secondary and higher education level, the risk of preterm births for illiterate mothers was 40% higher (aOR 1.41; 1.22–1.64), for literate mothers was 20% higher (aOR 1.21; 1.08–1.35) and mothers having basic level of education was 20%higher (aOR 1.17; 1.07–1.27). Similarly, the risk of preterm births among nulliparous mothers was 33% higher (aOR 1.33; 1.20–1.48) compared to multiparous mothers. Time for ANC visits was significantly associated with preterm births with those mothers going for ANC visits in the second trimester was 13% higher (aOR 1.13; 1.05–1.22) than those who went in the first trimester. The risk of preterm birth was 13% higher (aOR 1.13; 1.01–1.26) for mothers who had a history of smoking. Further, polluted fuel (aOR 1.26; 1.17–1.35), multiple deliveries (aOR 6.63; 5.16–8.52) and severe anemia during pregnancy (aOR 3.27; 2.21–4.84) were also significantly associated with preterm births. Female babies were at 1.18 times higher risk of being born preterm compared to males (aOR 1.18; 1.11–1.26) (Table 2).

**Table 2:** Multivariate analysis of factors associated with preterm birth (*n* = 49,898)

Variables	β – coefficient	***p***-value	aOR (95% CI)
**Age of mother**			
20- < 35 years	**Ref**		
< 20 years	0.234	**< 0.001**	1.26 (1.15–1.39)
> 35 years	0.180	0.082	1.20 (0.98–1.47)
**Education level of mother**			
Secondary and higher	**Ref**		
Illiterate	0.346	**< 0.001**	1.41 (1.22–1.64)
Literate	0.186	**0.001**	1.21 (1.08–1.35)
Basic education	0.152	**0.001**	1.17 (1.07–1.27)
**Parity of mother**			
Multipara	**Ref**		
Nullipara	0.285	**< 0.001**	1.33 (1.20–1.48)
Primipara	−0.013	0.812	0.99 (0.89–1.20)
**Time of first ANC visit**			
First trimester	**Ref**		
Second trimester	0.125	**0.001**	1.13 (1.05–1.22)
Third trimester	0.218	**< 0.001**	1.24 (1.12–1.38)
**ANC visits during pregnancy**			
≥4 visits	**Ref**		
< 4 visits	0.398	**< 0.001**	1.49 (1.38–1.61)
**Smoking**			
No	**Ref**		
Yes	0.121	**0.035**	1.13 (1.01–1.26)
**Type fuel**			
Clean	**Ref**		
Polluted	0.230	**< 0.001**	1.26 (1.17–1.35)
**Severe anaemia**			
No	**Ref**		
Yes	1.185	**< 0.001**	3.27 (2.21–4.84)
**Multiple delivery**			
No	**Ref**		
Yes	1.891	**< 0.001**	6.63 (5.16–8.52)
**Sex of the baby**			
Boy	**Ref**		
Girl	0.164	**< 0.001**	1.18 (1.11–1.26)
**Major malformation**			
No	**Ref**		
Yes	0.644	0.171	1.90 (0.76–4.78)

## Discussion

The study describes the incidence, risk factors and consequences associated with preterm births based on data available from 12 public hospitals across Nepal. The incidence of preterm births was found to be 9.8% among total births while it was 9.3% among live births. In a systematic review conducted with data available from 107 countries, the global preterm birth rate was reported at 10.6% [22] and a systematic analysis based on data available from 184 countries reported an estimated preterm births of 11.1% [1]. Studies conducted in the United States (9.62%) [23] and Australia (8.6%) [11] have also reported similar estimates. A previous study conducted in a tertiary hospital in Nepal reported at incidence of 8.1% for preterm births [24]. These findings suggest that Nepal’s preterm birth rate is in line with developed countries, suggesting an improvement over the national and global estimates.

This study looked at some of the potential risk factors for preterm births. The risk of preterm births was higher among mothers younger than 20 years. Several other studies have also reported linking both younger and older maternal age with preterm births [11, 25–30]. However, a study conducted in Bangladesh found women aged < 20 years to be protective for preterm, contrary to our findings [20]. Our study did not find any significant association with mothers aged 35 years and above.

The risk of preterm births was also higher among mothers with education lower than secondary level. Other studies have also shown similar associations related to lower education levels [14, 16, 20, 31]. This suggests that better educational status of mothers has a protective effect on birth outcomes. Further, mothers with a history of smoking had higher risk for preterm births. Other studies have also shown similar associations for preterm births [11, 32–34]. The risk of preterm was also higher for mothers who did not use clean fuel. A recent study conducted in China showed no significant association with the type of fuel used [35]. However, another study conducted in East India looking at the impact of the fuels in pregnancy outcomes showed a significant association for preterm births [36]. The variations could be due to the difference in sample sizes.

Our study showed that the risk of preterm was higher among nulliparous mothers. The findings are supported by previous studies [37, 38]. Babies born to mothers who seek ANC visits during second and third trimesters also had a higher risk of being preterm. Other studies have shown that seeking ANC visits later in pregnancies can increase the risks of preterm births [25, 27, 39]. Our study found that women who sought < 4 ANC visits during pregnancy were at higher risk of preterm birth. A study conducted in rural Gambia also showed higher risk though the findings were not significant [40]. Further, another study conducted by a Belgian team also found no substantial correlation between number of ANC visits and preterm birth but rather on the content and timing of care during pregnancy [39]. ANC visits should focus on improved screening of at-risk pregnant women together with the ability to treat and manage infections and provide dietary support and counseling services and further research is needed [41].

The risk of preterm births was also higher among women who had severe anemia during pregnancy. The finding is corroborated by other studies which showed severe anemia increased the risk of preterm births significantly [42–45]. Further, mothers who had multiple deliveries had a higher risk of having preterm births. A Korean study [46] and a cohort study in Bangladesh also showed similar findings [20]. However, a systematic review and meta-analyses assessing interventions aimed at preventing preterm births among twin pregnancies found that no interventions reduced the risk significantly [47].

The risk was also higher among women whose babies had major malformations although the association was not significant. However, previous studies have shown significant associations [48, 49]. One of the reasons could be the low numbers reported from our study. Sex of the child was associated risk with preterm births. Several studies have linked male babies to be at higher risk for preterm births [50]. However, a study showed no significant association between sex of the child and preterm births [51–53].

We also analyzed the consequences of preterm birth. Pre-discharge mortality was 11 times higher for preterm babies. Other studies have also reported similar findings [24, 54, 55].

There are some limitations in the study. The study did not analyze some of the risk factors (e.g. previous medical history, previous preterm births, cervical length, BMI etc.,) that have been discussed in the research articles as these data were either not collected in our study or they were underreported. Another limitation was that not all mothers took part in the interviews and for those who took part, the information might subject to recall bias. Further, we only analyzed pre-discharge mortality and therefore this study does not consider long term consequences. We did not collect data on other outcomes like birth-related injuries and other associated factors hence they are not reported in our study. One strength of this study is its’ large representative sample from 12 different hospitals. Hence, the results are a likely representation of the incidence of preterm births in Nepal.

## Conclusion

This study found that factors such as age of mother, literacy levels, smoking habits, delay in seeking ANC services, use of polluted fuel, multiple deliveries and severe anemia can provide as a risk factor for preterm birth.

The incidence of preterm in Nepal remains high. Based on the risk factors linked to preterm identified by this study, implementation of interventions focusing on improving women’s literacy, providing better access to clean fuel options, and improving lifestyle, may serve as a protective factor for preterm birth.

Further, improving access to ANC services allowing for better identification of complications, informed choices and safe delivery services can help in reducing risks and consequences associated with preterm births.

## Supplementary information

**Additional file 1.**

**Additional file 2.**

## Data Availability

The datasets analyzed for this study will be made available by the corresponding author upon request. Data collection forms (data extraction and exit interview) are provided along with the manuscript as Additional files 1 and 2.

## Abbreviations

LMICs: Low- and middle-income countries
ANC: Antenatal care
CSPro: Census and Survey Processing System
SPSS: Statistical Package for the Social Sciences
